# Radiological and Surgery Considerations and Alternatives in Total Temporomandibular Joint Replacement in Gorlin-Goltz Syndrome

**DOI:** 10.3390/diagnostics15091158

**Published:** 2025-05-02

**Authors:** Kamil Nelke, Klaudiusz Łuczak, Maciej Janeczek, Agata Małyszek, Piotr Kuropka, Maciej Dobrzyński

**Affiliations:** 1 Maxillo-Facial Surgery Ward, EMC Hospital, Pilczycka 144, 54-144 Wrocław, Poland; klaudiuszluczak@gmail.com; 2 Health Department, Angelus Silesius Academy of Applied Sciences in Wałbrzych, Zamkowa 4, 58-300 Wałbrzych, Poland; 3 Department of Biostructure and Animal Physiology, Wrocław University of Environmental and Life Sciences, Kożuchowska 1, 51-631 Wrocław, Poland; maciej.janeczek@upwr.edu.pl (M.J.); agata.malyszek@upwr.edu.pl (A.M.); 4 Division of Histology and Embryology, Department of Biostructure and Animal Physiology, Wrocław University of Environmental and Life Sciences, Cypriana K. Norwida 25, 50-375 Wrocław, Poland; piotr.kuropka@upwr.edu.pl; 5 Department of Pediatric Dentistry and Preclinical Dentistry, Wrocław Medical University, Krakowska 26, 50-425 Wrocław, Poland; maciej.dobrzynski@umw.edu.pl

**Keywords:** odontogenic keratocysts, OKCs, Gorlin-Goltz syndrome, temporo-mandibular joint, alloplastic TMJ reconstruction

## Abstract

Gorlin-Goltz syndrome (GGS) is also known as Nevoid basal cell carcinoma syndrome (NBCCS). In the most common manifestation, GGS is diagnosed based on multiple cysts in the jaw bones, namely OKCs (odontogenic keratocysts). Other features might include major and minor clinical and radiological criteria to confirm this syndrome. Quite commonly, BCCs (basal cell carcinomas), bifid ribs, palmar and plantar pits, and ectopic calcification of the falx cerebri can be found in the majority of patients. Currently, the mutation of the PTCH1 gene seems to be responsible for GGS occurrence, while the male-to-female ratio is 1:1. The following radiological study based on OPGs and CBCT confirmed multiple cystic lesions in jaw bones, confirmed to be OKCs in the histopathological evaluation with an occurrence of numerous skin BCC lesions. In cases of most oral OKC cystic lesions, either surgical removal, curettage, or enucleation with or without any bone grafting can be used with a good amount of success. Rarely, some stable bone osteosynthesis procedures have to be carried out to avoid pathological bone fractures after cyst removal. A special consideration should include the temporomandibular joint. TMJ surgery and the replacement of the joint with an alloplastic material can be performed to improve biting, chewing, proper mouth opening, and maintain good patient occlusion. The authors want to present how effective and simple a standard dental panoramic radiograph combined with CBCT is and how it is suitable for GGS detection. They also want to underline how a standard TMJ prosthesis can be used as an alternative to a custom-made prosthesis.

**Figure 1:**
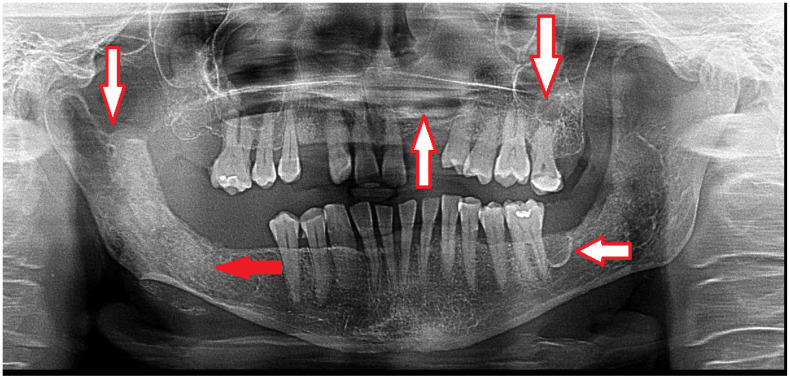
Gorlin-Goltz syndrome (GGS—Nevoid basal cell carcinoma syndrome, NBCCS) can be confirmed because of the occurrence of multiple odontogenic keratocysts (OKCs) in the mandible and maxillary bones (white arrows). This condition has many clinical and radiological characteristics. Because of the PTCH1 gene (a protein called patched-1) mutation, GGS can be present in different expression scales among patients. A standard OPG can easily detect some cystic lesions in jaw bones; however, CBCT (cone-beam computed tomography) is more accurate and precise in evaluating the shape, size, occurrence, and structure of each cyst. Radiologically, the occurrence of multiple cysts might suggest GGS; however, a histopathological evaluation should confirm the occurrence of OKCs after a biopsy (red arrow). Small cysts can be treated with a great success rate; however, some recurrences are possible [1]. In the CBCT, some cyst ranges and bone changes might be more visible, which would then influence the type of surgical approach that might be suitable for each case. In some cases, two-stage surgery consists of cyst decompression and the promotion of new bone growth. The presented panoramic radiograph also shows a significant mixed radiolucent–radiopaque lesion at the right condylar process and condylar head. A biopsy is always mandatory to confirm the final diagnosis and schedule the most adequate approach.

**Figure 2:**
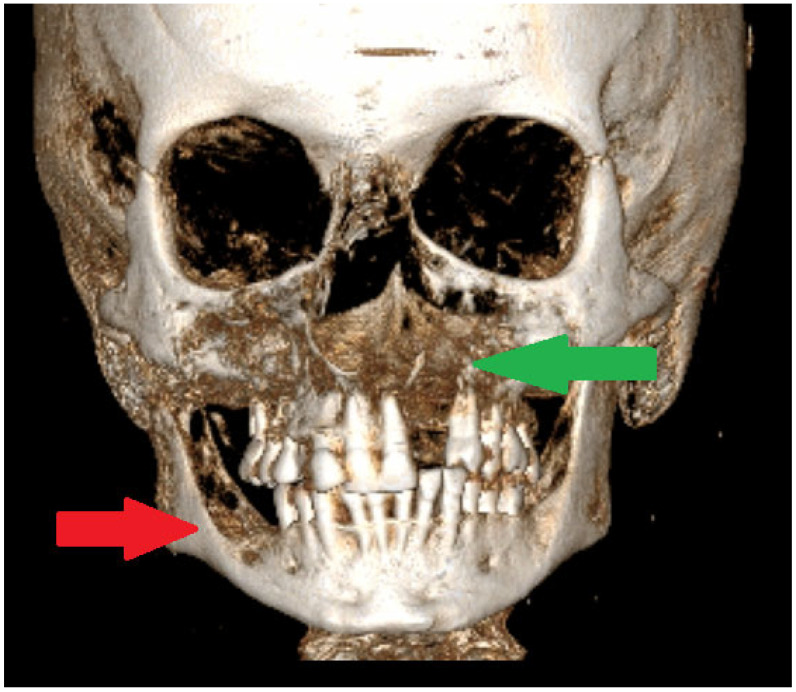
A CBCT-3D of jaw bones can greatly influence the scope of each surgical approach. Smaller bone lesions could be scheduled for a biopsy under local anesthesia. When, additionally, on facial skin, there are discoloured patches suggesting an early BCC (basal cell carcinoma), it is advisable to take two biopsies at the same time to improve the diagnosis of GGS. It is important to remember that GGS can be confirmed based on radiological and pathological evaluation and detailed clinical analysis. The following minor and major criteria for GGS confirmation were established [1,2,3]. The major criteria for NBCCS were as follows: two or more BCCs (basal-cell carcinomas) or one BCC before 20 years, the presence of two or more OKCs (odontogenic keratocysts), at least >3 cutaneous palmar or plantar pits, a 1st degree relative with NBCC (Nevoid basal cell carcinoma syndrome), and the presence of a medulloblastoma. On the other hand, the minor criteria for NBCCS were as follows: the presence of macrocephaly following the height adjustment; the presence of congenital orofacial defects such as frontal bossing, cleft palate, and hypertelorism; the presence of skeletal abnormalities such as syndactyly, pectus deformity or scapula defects, also the occurrence of a radiological abnormality such as fusion of the vertebras; hemivertebras; the bridging of Sella turcica and morphological defects of the hands and feet; the presence of an ovarian fibroma; and signs of bifid or fused ribs [1,2,3]. In many cases, during oral cavity, head, and neck evaluations, it is quite easy to see multiple cystic bone lesions, the presence of facial skin lesions/BCC, the presence of palatal defects and anomalies, and frontal bossing. In CBCT, it is easy to see a calcified falx cerebri and some disturbances with a high palate. The scope of each lesion might have different manifestations. In jaw surgery, special attention should be focused on extensive, expansile bone lesions causing visible bone disfigurement and bone asymmetry, and rarely occurring in the mandibular condyle head and temporomandibular area (TMJ). When TMJ structures are affected and the destruction of the joint occurs, some accompanying syndromes like joint pain, the inability to open the mouth, LMO-limited mouth opening, swelling and inflammation near the TMJ area, and problems with biting and chewing (red arrow). In elderly patients, this might also cause other problems related to prosthesis usage, overloading of the opposite side healthy joint, the growth of severe pain levels, and inflammation-related changes in the TMJ, which is where the OKC might spread [4,5]. Secondly, the problem is quite serious when a unicystic lesion of OKC has a polycystic appearance without borders and an expansive character causing bone asymmetry and cortical thickening, especially when present in the maxillary sinus, anterior maxillary bone, and alveolar process of the maxillary bone, or if any spread towards the nasal cavity (green arrow). Many approaches can be used to reduce the scope of resection, but the authors promote bone estectomy and allograft bone usage to strengthen the bones.

**Figure 3:**
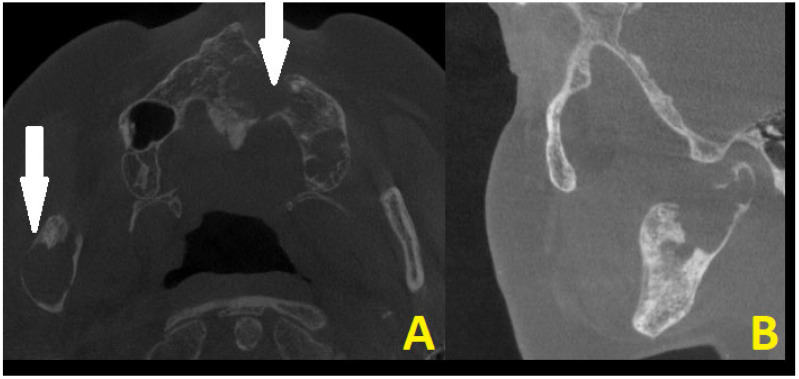
CBCT scans in axial projection (**A**) with maxillary destruction and sagittal (**B**) visualization of the cystic destruction of the right condylar process and condylar head. Cystic destruction of the left maxillary bone with an extra bone spread towards the lip and nasal cavity, and right mandibular ramus swelling with proliferation towards the condylar head is noted (white arrows). In surgical planning, the condition of the bone is very important for a good radiological bone evaluation, as well as for cortical bone estimation. Secondly, each surgery must maintain each patient’s quality of life (QOL). In the case of a 69-year-old patient, the most troublesome factors were related to an inability to fully open their mouth, the occurrence of transient pain, inflammation, the presence of right preauricular swelling, and the deterioration of chewing and biting ability. The first surgery step was used to remove all small lesions, fill them with allogenic bone grafts, and perform a right-sided coronoidectomy with cyst removal and decompression to prepare the right condyle for the second stage of surgery. The main troublesome course of surgery included the necessity to remove part of the bone TMJ structures affected by the disease and reconstruct the TMJ for the best possible outcome to enable the elderly lady to be able to properly open her mouth, eat, and speak. Because of this patient’s economic means and a great fear of a total TMJ artificial endoprosthesis, a decision was made to reduce both the costs and scope of surgery, meaning a reduction in the usage of many materials and foreign body components from a custom-made TMJ endoprosthesis. Secondly, since the OKC did not do any destruction within the condylar fossa, the articular disc and TMJ joint were healthy, so the usage of an acetabulum, namely the glenoid fossa alloplastic component, was not necessary. Planning a good CBCT is enough to plan the scope of bone resection and removal of all polycystic lesions from the right mandibular head, condyle, and part of the ramus. Because of the locally aggressive character of OKC, their extracortical spread, polycystic occurrence, and joint manifestation, a decision was made for more aggressive surgery with immediate joint reconstruction in the second stage of surgery when mouth opening was improved. Both surgeons (K.N., K.Ł.) decided to improve the overall mouth opening and excise all advanced aggressive OKC lesions at first, and then decide on the scope for TMJ surgery to improve occlusion and bite, and prepare for further patients’ prosthodontic treatment. No necessity for additional CT, MRI, or similar studies was needed.

**Figure 4:**
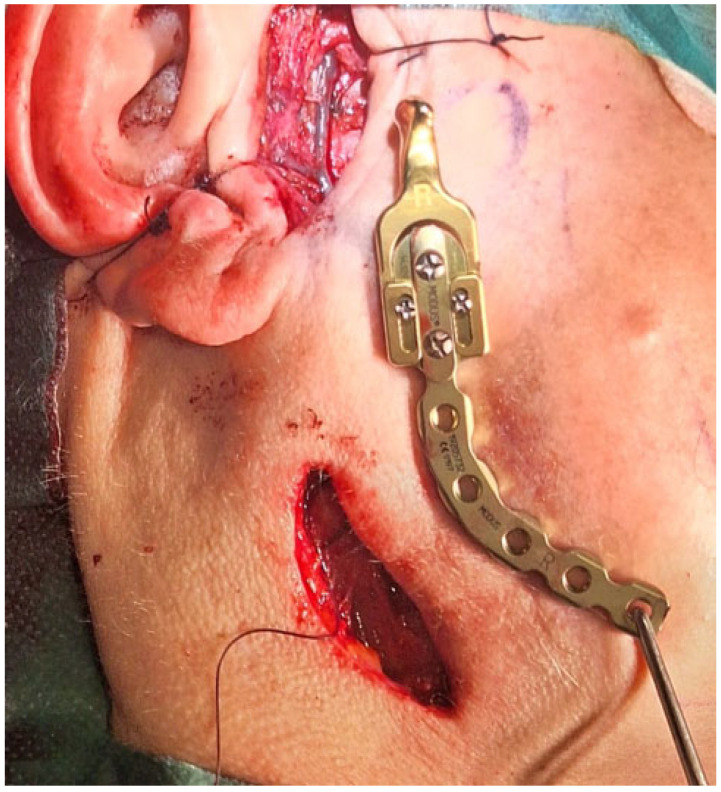
The surgical double approach consisted of the preauricular approach and the submandibular approach. The nasotracheal tube was sutured to the nasal septum and then retruded distally as per the tube placement algorithm [6]. A layer-by-layer preparation granted a good surgical view of the operated field with good protection of the facial nerve fibres. The TMJ standard prosthesis used was the Medartis 2.0 Modus System (Basel, Switzerland—right condylar head prosthesis (Titanium ASTM F67) with a carrier element, 2.0 mm four connecting screws screwed together with Medartis Reco plate 2.5, 2.5 mm connecting screws for proper mandibular vertical ramus height reconstruction, a stable position on the healthy part of the right mandibular ramus, and an angle free of OKC lesion. The pterygoid muscles were healthy and were sutured back to their initial position, in this case towards the titanium condylar head. The main lesion was located in the mandibular bone and had some cortical spread in the joint area. The articular disc and part of the capsule were healthy and not involved in the lesion, and because of that, fat grafting was not necessary. Any additional iliac crest bone grafting or fat was also not necessary. An additional four IMF (intermaxillary fixation) screws (Medartis, Basel, Switzerland), 2.0 system speed-tip 8/11 mm long, were used with elastic fixation. During the postoperative period, the patient was scheduled to use elastic rubbers on the IMF screws to stabilize the occlusion and surgery results. Currently, a custom-made individual TMJ prosthesis seems to be a good gold standard of treatment, especially in cases involving condylar disc damage, disruptions along the condylar fossa, and total joint resection. In some cases, such an approach with a standard, not custom patient-made, individual TMJ endoprosthesis solution is also quite effective, with fewer costs, good functional results, easy placement, no need for further glenoid fossa preparation, and the presence of just one metal alloy, the titanium. In this instance, the overall surgery outcome was very good, and the patient was satisfied with the results and the decreased necessity for any more advanced and costly procedures.

**Figure 5:**
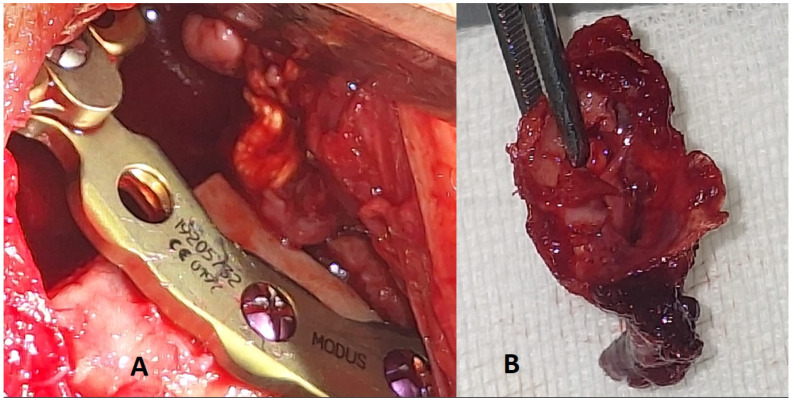
Excised condylar head with the OKC and final placement of the TMJ endoprosthesis. (**A**)—final position of the prosthesis with a gap, which did not require any additional bone or fat grafting to cover it. (**B**)—excised specimen of the condylar head and the remnants of the condylar bone. A TMJ alloplastic joint reconstruction in Gorlin-Goltz syndrome is a very rare finding. The usage of standard TMJ prosthesis is a valuable alternative approach.

**Figure 6:**
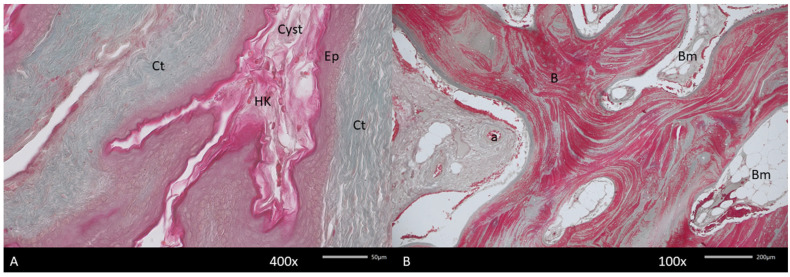
Histopathological picture of the keratocyst (Cyst). (**A**). Visible hyperkeratinized (HK) stratified epithelium (Ep) lining keratocyst wall surrounded by dense connective tissue (Ct). The deeper fragment of the cyst contains new bone. (**B**). Arranged in a multidirectional form, containing bone marrow (Bm). In the connective tissue, numerous blood vessels, both arteries (a) and veins, are noted. Mallory stain.

**Figure 7:**
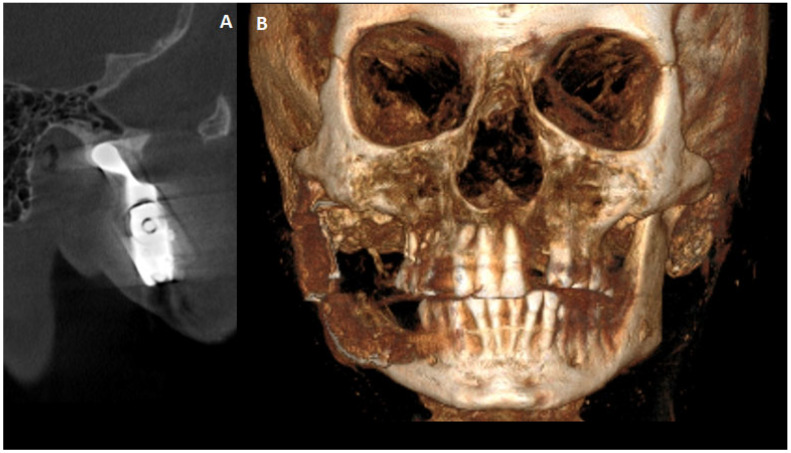
A final 12-month postoperative CBCT to evaluate the final results from surgery. (**A**)—CBCT sagittal scan with good TMJ position in the central spot of the glenoid fossa. (**B**)—CBCT-3D reconstruction with good bite, proper anterior facial height, and stable occlusion. In some cases, procedures concerning total temporomandibular joint replacement surgery (TMJR) greatly influence patients’ quality of life (QOL) [7]. Those aspects are related to improved mouth opening, the ability to bite and chew, restored balanced occlusion, and improved oral food intake. Since the concept of standard-made TMJ endoprosthesis is less common than it has been previously, it is worth knowing that in this case of Gorlin-Goltz syndrome, this approach is still worth considering. For example, if any OKC recurrence or new OKC cystic lesion near the TMJ prosthesis is found, it is always possible to change the device for a custom-made one or change the plating shape and length to keep the standard one in place. All TMJ alloplastic devices have many benefits that have been discussed widely in the literature.

## Data Availability

The datasets used and/or analyzed during the current study are available from the corresponding author upon reasonable request.
